# An Essay on the Tongue in Functional Derangement of the Stomach and Bowels, and on the Appropriate Treatment, &c.

**Published:** 1847-04-01

**Authors:** 


AN ESSAY ON THE TONGUE IN FUNCTIONAL DERANGEMENT OF THE STOMACH AND BOWELS, AND ON THE APPROPRIATE TREATMENT; ALSO THE TONGUE'S ASPECT IN ORGANIC DISEASE OF THE LUNGS AND HEART. By *Edward Williams*, M.D., Senior Physician to the Essex and Colchester Hospital. Second Edition, 8vo. pp. 236. Simpkin and Co. and Renshaw. London, 1847.

THE following extract from the Author's Preface gives, if we mistake not, the very pith and kernel of his elaborate researches.

"It was after much reflection that we began arranging the materials for the Essay, nor was it without considerable labour in selecting from the case-books those instances in which the tongue's aspect was described, and by the avoiding those in which the tongue's appearance might have been influenced by medicaments, that we were enabled to shape our proceedings; however, 'in all labour there is profit,' and the tongue presented itself under two principal aspects, namely, when the papillæ were developed, and when they were not observable.

"In pursuing the subject, by arranging the cases in a tabular form, it became apparent that when the papillæ, especially the filiform and tuberose, were prominent or florid, that the gastric symptoms were the most prevalent, and this circumstance led to the examining the symptoms attending a development of the different orders of the papillæ. A careful analysis of these cases established the inference, that the stomach was specially affected when the filiform or tuberose papillæ were developed; hence 'the tongue of gastric functional derangement.'

"A like review of the remaining cases led to the conclusion, that disturbance of the intestinal canal was accompanied with certain appearances of the tongue, the papillæ not being observable, and thus originated the 'tongue's aspect in functional derangement of the intestines.'" P. vi.

Even if we had space to spare, it would have been utterly impossible to give the reader anything like a correct idea of the contents of Dr. Williams's volume. The details communicated are surprisingly minute, and none but a most zealous and pains-taking observer could have had the patience to record and compare such a host of particulars. Those who feel an interest in all the curious niceties of symptomatology and diagnosis in reference to an individual organ of the body, cannot but be gratified with our author's work.

